# Telemedicine among Adults Living in America during the COVID-19 Pandemic

**DOI:** 10.3390/ijerph20095680

**Published:** 2023-04-28

**Authors:** Man Hung, Monica Ocampo, Benjamin Raymond, Amir Mohajeri, Martin S. Lipsky

**Affiliations:** 1 College of Dental Medicine, Roseman University of Health Sciences, South Jordan, UT 84095, USA; mocampo283@student.roseman.edu (M.O.); braymond074@student.roseman.edu (B.R.); amohajeri@roseman.edu (A.M.); mlipsky@pdx.edu (M.S.L.); 2 Division of Public Health, University of Utah, Salt Lake City, UT 84108, USA; 3 Department of Educational Psychology, University of Utah, Salt Lake City, UT 84109, USA; 4 College of Social Work, University of Utah, Salt Lake City, UT 84112, USA; 5 Department of Veterans Affairs Medical Center, Salt Lake City, UT 84148, USA; 6 Institute on Aging, Portland State University, Portland, OR 97201, USA

**Keywords:** telemedicine, telehealth, COVID-19, pandemic

## Abstract

Background and Objectives Telemedicine can expand healthcare access to populations, but relying on technology risks a digital divide. Therefore, it is important to understand who utilizes telemedicine. This study explores telemedicine usage across socio-demographic groups in the United States during COVID-19. Methods Data came from the Household Pulse Survey (HPS) between 14 April 2021, to 11 April 2022. HPS is a rapid online response survey that assesses household experiences during COVID-19. We calculated descriptive statistics and used cross-correlation to test each pair of the time series curves. Results High school graduates used the least telemedicine (20.58%), while those with some college (23.29%) or college graduates (22.61%) had similar levels, and those with less than a high school education fluctuated over time. Black people had higher levels of use (26.31%) than Asians (22.01%). Individuals with disabilities (35.40%) used telemedicine more than individuals without disabilities (20.21%). Individuals 80 years or over (27.63%) used telemedicine more than individuals 18 to 29 years old (18.44%). Cross-correlations for the time series pairs across demographics revealed significant differences in telemedicine use for all demographic groups over time. Conclusions Overall, elderly, Black people, individuals with some college, and persons with disabilities report higher levels of telemedicine use. Telemedicine may improve healthcare access post-pandemic, but more research is needed to understand factors that drive differences among groups.

## 1. Introduction

The American Telemedicine Association defines telemedicine as the “use of medical information exchanged from one site to another via electronic communications to improve a patient’s clinical health status” [1]. The term telemedicine is often confused and used interchangeably with a related, though distinct, term telehealth. While telehealth may include both clinical and non-clinical services, the American Academy of Family Physicians classifies telemedicine as the use of technology to deliver care at a distance, over a telecommunications infrastructure that enables the physical and virtual transmission of components such as voice, video, and data between a patient at an originating (spoke) site and a physician or other licensed healthcare providers and mental health therapists to practice at a distance (hub) site. In contrast, telehealth refers to a broader term and includes the use of an array of electronic and telecommunication technologies and services that support distance health care delivery and services [2] such as phone, e-mail, and remote patient monitoring. This study focused on telemedicine or when providers use video connections to conduct the equivalent of an in-person visit.

Telemedicine began in the United States (US) during the mid-twentieth century and by the 1980s became a tool to improve access to remote, rural communities that lacked medical services [3]. By the 1990s, the emergence of the internet created a platform that broadened the application of telehealth to include the transmission of X-rays, electrocardiograms, scans, and real-time audio and video interactions [4]. As more Americans accessed the internet and used smartphones and/or computers, telemedicine continued to expand [4,5]. By 2017, about three-quarters of US hospitals interacted with patients through the use of video and other technology [6].

The COVID-19 pandemic and lockdowns generated unparalleled economic and social disruption. The Centers for Disease Control and Prevention recommended physical distancing to reduce coronavirus spread and telemedicine offered an opportunity to mitigate transmission to patients, providers, and staff. As a result, it is not surprising that telemedicine visits are about 40 times more frequent than before the pandemic [7]. Telemedicine use which includes evaluation and management visits for general medicine, dental medicine, and several other high-volume specialties spiked to 32% in April 2020 after COVID-19 was declared a public health emergency [8]. After this initial spike, telemedicine use stabilized to between 13 and 17% of office and outpatient visits across all specialties. Its expanded use makes it important that health care providers become competent using telemedicine to provide patient care [9], and as Hyder et al. noted, telemedicine is “critical to ensuring public health and is poised to become a reliable and acceptable method of seeking care for many conditions” [10].

Despite the growth and greater acceptance of telemedicine as a mainstream modality, concerns about its use remain. Although telemedicine offers the promise of improving access to rural communities and other populations facing access barriers, a reliance on technology also creates a risk of a digital divide that potentially widens the gap in health disparities rather than narrowing them. The absence of technology, digital literacy, and reliable internet coverage are some of the barriers that prevent patients from accessing some forms of telemedicine, such as video [11]. These barriers are more common among low-income populations that have been disproportionately affected by COVID-19. In the United States, a quarter of adults with incomes below $30,000 a year do not own a smartphone [12]. Additionally, a large proportion of adults with lower incomes do not have home broadband (43%) or a laptop (41%) [12]. There is evidence that low-income, non-English-speaking, individuals with disabilities and older patients are more likely to encounter barriers to accessing telemedicine care [13,14], which could exacerbate existing inequities as telemedicine becomes more widespread [15]. In contrast, females may be more likely to engage in electronic health activities [16] than males, with African American males being among the least intense users of the internet [17].

Given this, we sought to further investigate for the possible presence of inequities in telemedicine use more broadly across the US. To identify the possible presence and scope of a telemedicine digital divide, a first step is a better understanding of who utilizes telemedicine. Using a national database, this study sought to describe telemedicine use and explore what differences, if any, exist among different demographic groups. A second aim was to examine trends in use over a year. Understanding whether differences in groups exist can guide strategies to avoid digital disparities in telemedicine treatment delivery.

## 2. Methods

### 2.1. Data Source

The data for this study came from the Household Pulse Survey (HPS) adult sample from 14 April 2021 to 11 April 2022 [18]. HPS is a 20 minute rapid-on-line response survey of adults aged ≥18 years old led by the US Census Bureau in collaboration with the National Center for Health Statistics, the Health Resources and Services Administration’s Maternal and Child Health Bureau and other federal agencies to assess household experiences during the COVID-19 pandemic. The survey was administered online and measures core demographic characteristics and includes questions related to COVID-19 vaccinations, food sufficiency, household spending, household energy expenditures and consumption, housing security, physical and mental health, and rental assistance from state and local governments that are designed to provide data about the impact of COVID-19. In addition, the survey contained questions about telemedicine use.

HPS data collection began on 23 April 2020, and is collected in different time periods. This study assessed measures at monthly intervals from April 2021 to April 2022 with a total sample size of 989,712 over the entire time. To develop a nationally representative survey sample, HPS uses the US Census Bureau’s Master Address File to obtain a nationally representative sample of over one million housing units to yield about 70,000 respondents who answered the online questionnaire [19]. It also employs weighting procedures to account for differential nonresponse and to match Census Bureau estimates of the population by demographics at three different geographical levels. The first level of geography is for the 15 largest Metropolitan Statistical Areas in the US. The second level is state-level estimates for each of the 50 states plus the District of Columbia, and the final level is national-level estimates [19]. The HPS survey was conducted via an internet questionnaire, with invitations for individuals to participate sent via email and text message using email addresses and cell phone numbers obtained from the Census Bureau Master Address File Data. More details about the HPS can be found at: https://www.census.gov/data/experimental-data-products/household-pulse-survey.html (accessed on 21 April 2023).

### 2.2. Ethics Statement

HPS was a voluntary survey study conducted by the US Census Bureau under the authority of Title 13, United States Code, Sections 8(b), 182 and 193, and approved by the US Office of Management of Budget (OMB approval number 0607-1013). A Waiver of Documentation of Informed Consent from the participants was approved by OMB. It is allowed by 45 CFR 46.117. Waivers can be approved for minimal risk research such as surveys/interviews conducted via telephone or online [20,21]. By using the United States Government computer network, participants consent to the collection, monitoring, recording, and use of their information for any lawful government purpose [22].

### 2.3. Study Variables

HPS adult respondents’ demographic characteristics were the independent variables, and included education level (Less Than High School, High School Diploma or General Education Development, Some College or Associate Degree, Bachelor Degree or Higher), state residence (individual states in the US), disability status (Disability, No Disability), age (18–29 years, 30–39 years, 40–49 years, 50–59 years, 60–69 years, 70–79 years, 80 years and above), self-identified gender (Male, Female), and race/ethnicity (Non-Hispanic Asian, Non-Hispanic Black, Non-Hispanic White, Non-Hispanic Other Races or Multiple Races). The dependent variable was telemedicine use, which was represented by the following question—At any time in the last 4 weeks, did you have an appointment with a doctor, nurse, or other health professional by video or by phone? (Yes, No). Please only include appointments for yourself and not others in your household.

Educational level and age were coded as ordinal variables and all other variables were coded as nominal variables.

### 2.4. Statistical Analyses

The percentages of telemedicine use for each demographic variable were calculated and a time series graph was plotted for the demographic variables. To examine the trends in telemedicine use across demographic groups, cross-correlation to test each pair of the time series curves was used. Cross-correlation measures similarity between two time series and allows the identification of lags to assess the degree that one time series correlates with another time series. It is appropriate for testing the trends of a variable across time among groups. Cross-correlations and their associated 95% confidence level were calculated and reported for the time series of each demographic variable. The results from the cross-correlation functions of the time series determine whether two time series are connected and whether the movement in one time series precedes or follows the movement of another time series at various lags. When the correlation coefficient of two time series is within the 95% confidence limit, the correlation is deemed non-statistically significant.

## 3. Results

**Figure 1:**
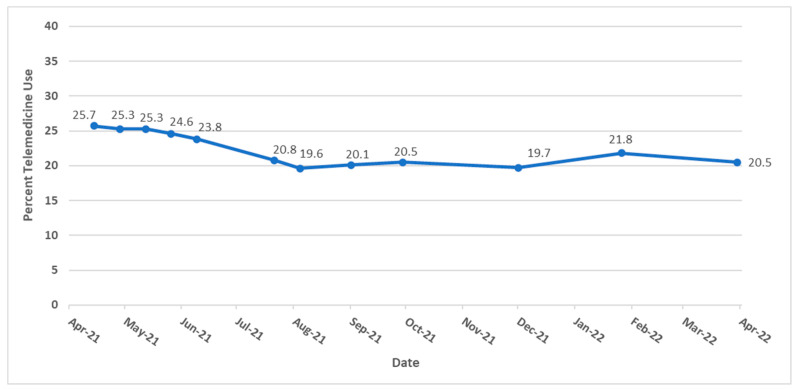
Trends of telemedicine use from 2021–2022 in the United States.

**Figure 2:**
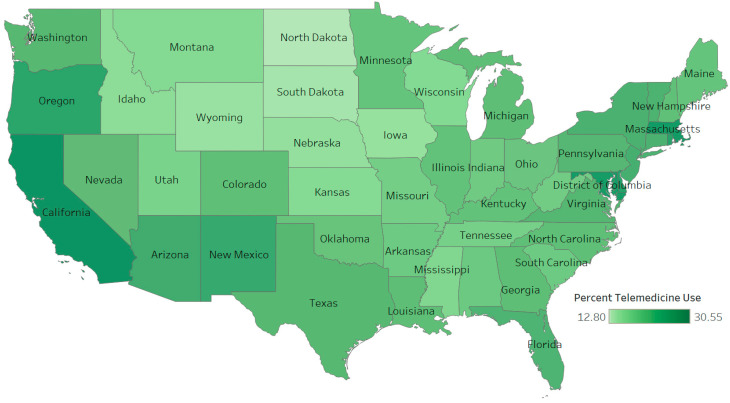
Telemedicine use across the United States.

Table 1 displays the sample size for each data collection period and response rate over each period. Sample sizes ranged from 49,230 to 71,848 representing response rates from 5.4% to 7.9%. From April 2021 to April 2022, the average daily level of telemedicine usage in the US ranged from 19.6% to 25.7% (mean = 22.31%, std. dev. = 2.43%) with a steady decrease in use over the study period (Figure 1). Among the 50 states and the District of Columbia, North Dakota utilized telemedicine the least (mean = 12.80%, std. dev. = 2.47%) while District of Columbia residents accessed telemedicine services the most (mean = 30.55%, std. dev. = 6.70%). Figure 2 shows a map of telemedicine use across the US, where states with darker colors experienced a higher percentage of telemedicine usage.

**Figure 3:**
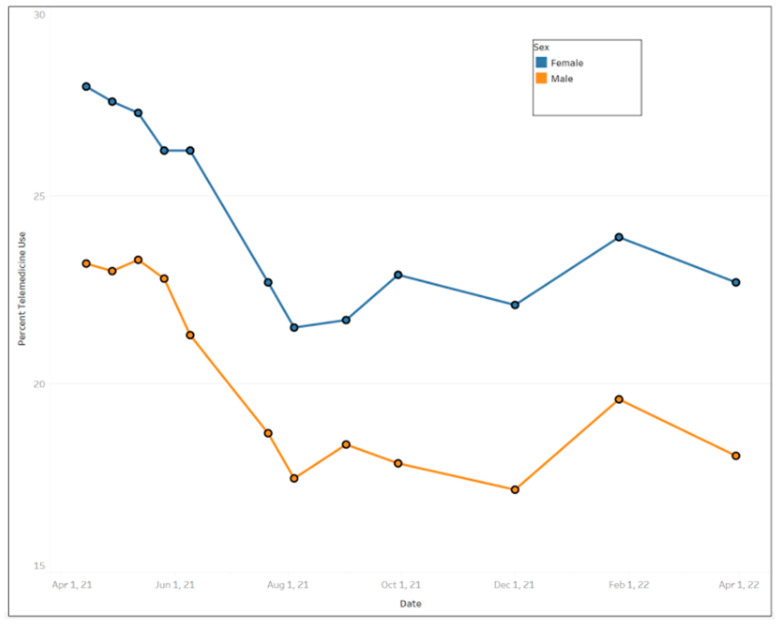
Trends of telemedicine use from 2021–2022 across gender.

**Figure 4:**
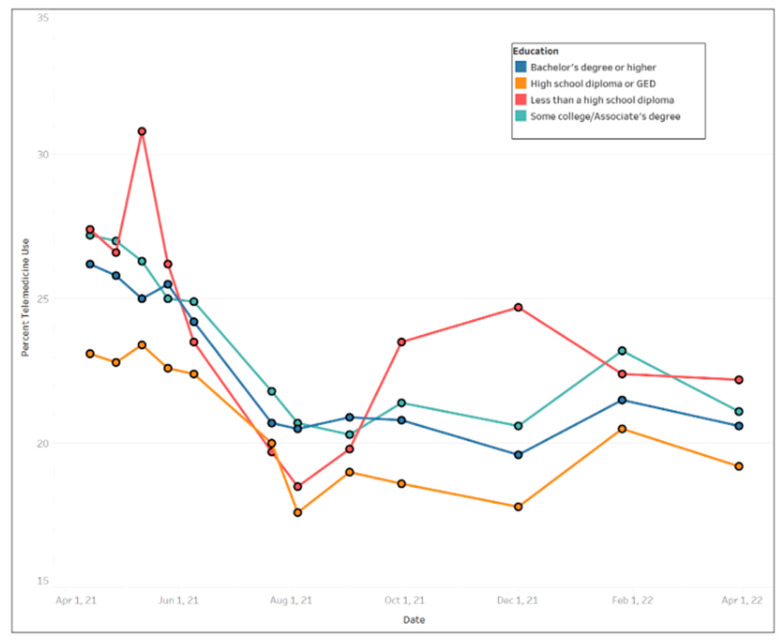
Trends of telemedicine use from 2021–2022 across education levels.

**Figure 5:**
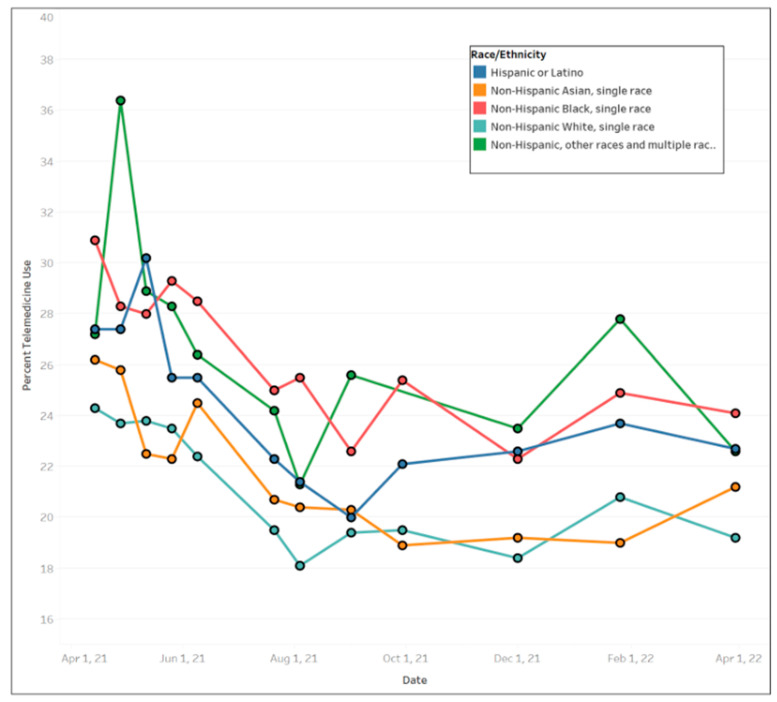
Trends of telemedicine use from 2021–2022 across race and ethnicity.

**Figure 6:**
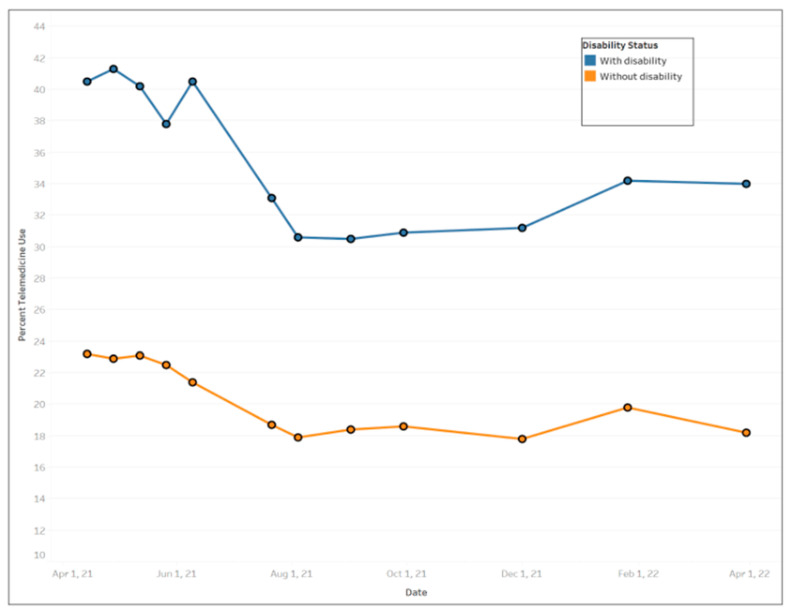
Trends of telemedicine use from 2021–2022 across disability status.

**Figure 7:**
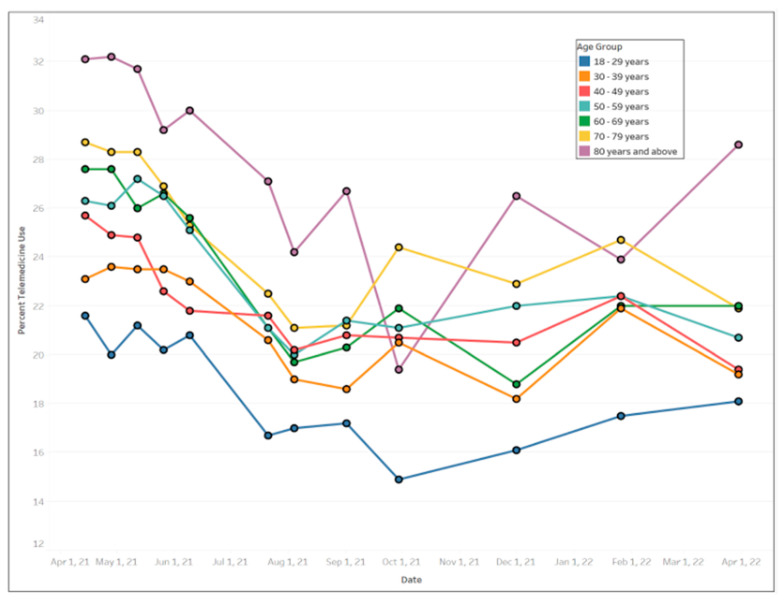
Trends of telemedicine use from 2021–2022 across age groups.

In general, females used more telemedicine services than males (Figure 3), with average use of 24.77% (std. dev. = 2.66%) and 20.56% (std. dev. = 2.35%), respectively. Across educational levels, individuals who had a high school diploma or general education development (GED) used telemedicine the least (mean = 20.58%, std. dev. = 2.39%) compared to other educational groups. Those with less than a high school education exhibited the largest fluctuation in telemedicine utilization over the time period (Figure 4) ranging from a high of 30.8% between 12 May and 24 May 2021 to a low of 18.5% between August 4 and August 16, 2021. Figure 5 shows that Black people used telemedicine more (mean = 26.31%, std. dev. = 4.99%) than other ethnic groups, and Figure 6 reveals persons with disabilities (PWDS) used telemedicine substantially more (mean = 35.40%, std. dev. = 4.58%) than individuals without disability (mean = 20.21%, std. dev. = 2.25%) throughout the study period. Usage for PWDS peaked at nearly 42% early in the study period but leveled off to 32% by 1 April 2022. Additionally, older individuals aged 80 years or over used telemedicine (mean = 27.63%, std. dev. = 2.53%) more often than younger individuals aged 18 to 29 years old (mean = 18.44%, std. dev. = 2.42%) (Figure 7). 

**Figure 8:**
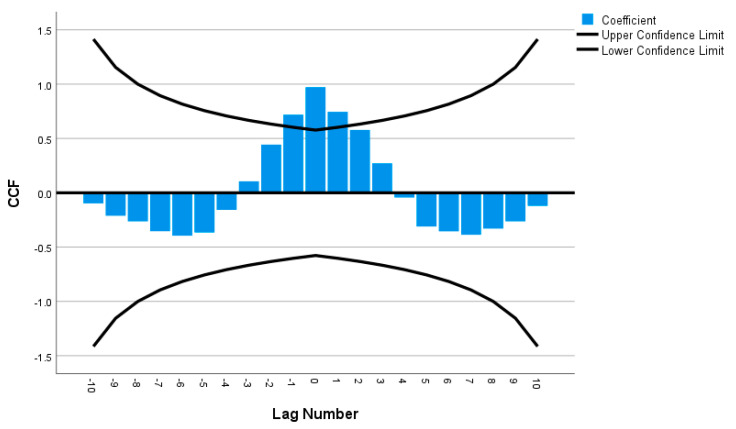
Cross-correlation as a function of lag number in female versus male’s time series.

**Figure 9:**
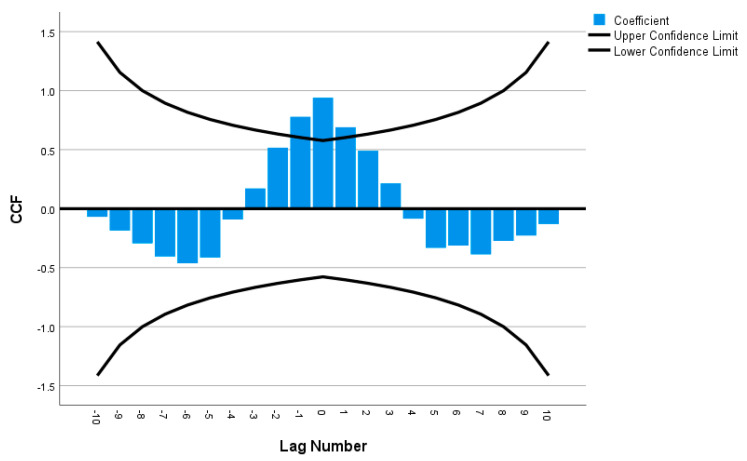
Cross-correlation as a function of lag number in disability versus without disability’s time series.

**Figure 10:**
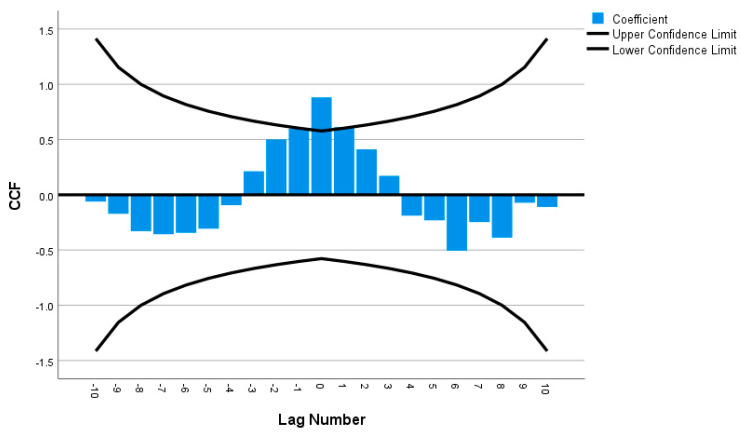
Cross-correlation as a function of lag number in individuals aged 18 to 29 years old versus aged 80 years or over’s time series.

Cross-correlations were computed for all of the time series pairs across demographics (such as female versus male, with a disability versus without disability, 80 years or over versus 18 to 29 years old, etc.); however, none of the time series pairs were within the 95% confidence intervals, indicating that there were significant differences in telemedicine use between all of the demographic groups over time. For instance, in Figure 8, the male and female time series were not strongly correlated since the cross-correlation was not statistically significant at most of the lag numbers; only lag numbers −1, 0 and 1 were statistically significant (i.e., falling outside of the 95% confidence interval), supporting the results that females’ usage of telemedicine was indeed more than males’ usage (Figure 3). Figure 9 also reveals weak correlation between the with disability and without disability time series, with only lag numbers −1, 0 and 1 being statistically significantly related, confirming that persons with disability used more telemedicine than persons without disability (Figure 6). In examining time series of individuals aged 18 to 29 years old versus individuals aged 80 years or over, it was found that only one lag number (at 0) had significant correlation, but the rest did not (Figure 10), demonstrating that the younger individuals used less telemedicine than older individuals at one of point in time (Figure 7).

## 4. Discussion

Telemedicine became a frontline strategy to mitigate the COVID-19 pandemic’s impact on the health of individuals and the country [23]. It offered a treatment option that provided access to care and by avoiding in-person visits reduced the risk of disease transmission for both patients and providers. This study explored adults’ usage of telemedicine across socio-demographic groups in the US during the COVID-19 pandemic. It revealed significant variations in telemedicine use based on demographic characteristics with the groups who reported the greatest use consisting of those with some college education or less than a high school diploma, disabled individuals, the elderly, non-Hispanic Black peopleand non-Hispanic other races. Since HPS collects data weekly and the survey results fluctuated over the study period, a time series cross correlation was used to examine patterns and trends in the data over the study period. This analysis demonstrated that females, PWDS and older adults used telemedicine significantly more than males, persons without disabilities and younger adults, respectively.

Before COVID-19, the use of telemedicine grew rapidly and from 2010 to 2017 the percentage of hospitals using telehealth increased from 35% to 76% [6]. Even so, a 2019 study found that only about 1 in 10 individuals reported using telehealth, and less than one in five were aware that their health system offered telehealth as an alternative to an in-person visit [24]. However, with lockdowns and social distancing, telemedicine (or telehealth in general) sometimes became the most viable option for non-emergency care. The results of our study showed that more than one in five adults reported using telemedicine within the past four weeks which suggests a broader awareness and acceptance by both providers and patients, consistent with other studies reporting an expansion and overall acceptance of telemedicine during the COVID-19 pandemic [25,26,27,28].

Encounters declined by about 5% over the study period, which may reflect individuals becoming vaccinated and an easing of restrictions, facilitating better access to in-person visits. Nonetheless, telemedicine usage remained much higher than before COVID-19. While the desire to mitigate SARS-CoV-2 spread and protect patients stimulated wider use of telemedicine [29], its expanded use offers post-pandemic opportunities to improve access, reduce costs and improve outcomes [30].

The shift to telemedicine during the pandemic raised expectations that telemedicine could narrow gaps in health inequities but with this hope came concerns that barriers to access and utilization of telemedicine among vulnerable groups might increase disparities. Those at greatest risk for falling behind included older adults who might have less access and less familiarity with technology, PWDS, minorities, and those with less education.

The results from HPS survey data suggest that telemedicine may help to bridge gaps by easing barriers such as travel, missing work or school, the need to arrange for childcare, and accessing specialty services. PWDS exhibited the highest usage, but similar to other groups the usage decreased over time. Since PWDS are among the groups disproportionally affected by COVID-19, the finding that about 40% answered yes to using telemedicine was reassuring. However, the study did not explore how different types of disability affected telemedicine use. Those with certain types of disabilities might have benefited greatly, while others with disabilities such as hearing or cognitive impairment that make telemedicine access more challenging than an in-person visit [31,32] might report different levels of utilization. The high level of usage highlights the importance of research that explores the effectiveness and satisfaction with telemedicine among PWDS.

Older adults are often associated with telemedicine inequality because of limited internet access, poorer internet skills, issues with computer literacy, and lower acceptance of technology. A 2020 retrospective medical record review at a large academic health system found that older age was independently linked to lower telemedicine use [33]. In contrast, our results found that older adults were more likely to have engaged in a telemedicine visit than younger adults. One reason may be that older adults are more likely to seek care because they have more chronic conditions than younger individuals who might view their health issues as less urgent. However, elderly patients, who engage in telemedicine report they value the convenience and express high levels of patient satisfaction and acceptance [34]. It may be after experiencing a telemedicine visit older adults felt comfortable continuing to schedule telemedicine visits.

Individuals with higher education usually have more access to technology and internet services [35] and our finding that those with a high school diploma or a GED used telemedicine less than those with more education is not surprising. One interesting finding was that those with less than a high school education exhibited the largest fluctuation in telemedicine utilization showing the highest peak in usage. The reason for this fluctuation is not clear but even though this group might face technology related issues these may be less of a barrier than in the past. According to the Pew Research Center, 90% of Americans use the internet, 85% of Americans own a smartphone, and about 75% own desktop or laptop computers. South Dakota and several other midwestern states reported less use of telemedicine than other states and this may be due to fewer pandemic related restrictions in many of these states. Several of these states also have higher percentages of rural residents and lower rates of telemedicine may be related to internet access barriers for those living in rural communities.

Another key finding was that Black people used telemedicine more than other groups. Since minorities may experience a digital divide, this finding is reassuring. While some have found less use by individuals of color [36,37], our results align with other studies such as a study exploring telemedicine use in California elementary schools which also found that Black students exhibited the highest rate of use and a Pew Research Survey which similarly found that Black individuals exhibited higher usage [38,39]. Our study did not explore why Black people used telemedicine more, but one reason may be that they perceived COVID-19 as a greater threat [40] because of its disproportionate impact on their communities. African Americans also experience more chronic health conditions [41]; thus, pandemic restrictions might have led them to use telehealth more to maintain their ongoing care. One potentially positive consequence is that the greater use of telemedicine during the pandemic may create an opportunity for reducing health disparities post-pandemic.

Even though women tend to adopt new technological applications later than men, our study found that women accessed telemedicine services more than men [42], a finding consistent with other studies [43]. This is not surprising since females tend to have more medical visits than males and were more likely to use telemedicine pre-pandemic [44]. Women are more likely to rate telemedicine care as excellent compared to men [45] and they may be more likely to be repeat users of telemedicine than men. 

### Limitations

There are several limitations to this study. One is that this study is based in the US and may not be applicable to other countries, especially those with less access to broadband and communication technologies. HPS only recruits households that have an email address or a cell phone number which could bias results. Despite using weighted data to mitigate non-response bias, internet-based surveys can introduce bias since individuals who are more comfortable with technology are more likely to respond to online surveys which can limit its generalizability to other populations. Additionally, HPS does not control for baseline differences in health status or overall rates of health care use and some differences among groups with more telemedicine use may simply reflect groups that are more likely to use any health care. Additionally, the response rate for HPS is lower than other federal databases, and it ranges from about 1 to 10% depending on the week [46]. Responses are self-reported and are subject to recall bias. However, direct feedback may capture data that other sources such as claims data miss and while the rapid response of HPS is a strength, the real-time nature of the survey provides limited time to identify and fix processing errors. Finally, telemedicine use peaked in 2020 and this study used data from 2021 and 2022.

## 5. Future Research

The COVID-19 pandemic stimulated the growth of telemedicine but also raised ethical and legal concerns [47]. While this study identified disparities in telemedicine use, it also suggests the need for further research to identify the cause for these disparities, their impact on patient outcomes, effect on equitable access, and how inequalities may affect patient satisfaction and safety. It will also be important to examine how provider type may affect demographic disparities. Post-pandemic changes in telemedicine regulations [48] and reimbursement [49] post-pandemic may also affect demographic disparities and merit future investigation. As Christie [50] notes a “One Size Fits All” approach to telemedicine may not serve those less tech savvy or patients with multiple co-morbid conditions. 

### Implications for Practice

The pandemic created a natural experiment to explore the expansion of telemedicine. Our findings suggest that in addition to its role to help manage a healthcare crisis it could potentially become a mainstream tool to overcome barriers to care for vulnerable groups such older adults, minorities, and PWDS provided that issues such as reimbursement and regulatory and technological challenges can be adequately addressed. 

## 6. Conclusions

Our results show differences in telemedicine utilization with older adults, Black people and females, and persons with disabilities reporting higher levels of use. These findings suggest that telemedicine may not exacerbate inequalities in care among certain vulnerable groups and may offer an opportunity to improve healthcare access post-pandemic. However, more research is needed to understand the factors that drive differences among groups.

## Figures and Tables

**Table 1:** Sample sizes and response rates across data collection periods.

Data Collection Period	Sample Size	Response Rate
30 March–11 April 2022	54,830	6.0%
2–14 March 2022	71,848	7.9%
26 January–7 February 2022	66,542	7.2%
29 December 2021–10 January 2022	67,259	7.2%
1–13 December 2021	53,078	5.8%
29 September–11 October 2021	49,230	5.4%
15–27 September 2021	51,652	5.6%
1–13 September 2021	54,950	6.0%
18–30 August 2021	59,705	6.5%
4–16 August 2021	59,362	6.5%
21 July–2 August 2021	55,699	6.1%
23 June–5 July 2021	52,838	6.3%
9–21 June 2021	54,267	6.4%
26 May–7 June 2021	56,436	6.7%
12–24 May 2021	58,286	6.8%
28 April–10 May 2021	62,895	7.4%
14–26 April 2021	54,835	6.6%

## Data Availability

Data are available at https://data.cdc.gov/NCHS/Telemedicine-Use-in-the-Last-4-Weeks/h7xa-837u (accessed on 5 July 2022).

## References

[1] Miller K. What Is Telemedicine, Exactly? Shape. Published 18 March 2020. https://www.shape.com/lifestyle/mind-and-body/what-is-telemedicine.

[2] AAFP Advocacy Focus: Telehealth and Telemedicine. Telehealth and Telemedicine. Published 2019. https://www.aafp.org/advocacy/advocacy-topics/health-it/telehealth-telemedicine.html.

[3] Nesbitt T.S. (2012). The Evolution of Telehealth: Where Have We Been and Where Are We Going?. The Role of Telehealth in an Evolving Health Care Environment: Workshop Summary.

[4] Kichloo A., Albosta M., Dettloff K., Wani F., El-Amir Z., Singh J., Aljadah M., Chakinala R.C., Kanugula A.K., Solanki S. (2020). Telemedicine, the current COVID-19 pandemic and the future: A narrative review and perspectives moving forward in the USA. Fam. Med. Community Health.

[5] Totten A.M., McDonagh M.S., Wagner J.H. (2020). The Evidence Base for Telehealth: Reassurance in the Face of Rapid Expansion during the COVID-19 Pandemic.

[6] (2019). Fact Sheet: Telehealth. American Hospital Association. https://www.aha.org/system/files/2019-02/fact-sheet-telehealth-2-4-19.pdf.

[7] Bestsennyy O., Gilbert G., Harris A., Rost J. (2021). Telehealth: A Quarter-Trillion-Dollar Post-COVID-19 Reality? McKinsey & Company. https://www.mckinsey.com/industries/healthcare-systems-and-services/our-insights/telehealth-a-quarter-trillion-dollar-post-covid-19-reality.

[8] Eyrich N.W., Andino J.J., Fessell D.P. (2021). Bridging the Digital Divide to Avoid Leaving the Most Vulnerable Behind. JAMA Surg..

[9] Hasselfeld B.W. Benefits of Telemedicine. Johns Hopkins Medicine, Published 2020. https://www.hopkinsmedicine.org/health/treatment-tests-and-therapies/benefits-of-telemedicine.

[10] Hyder M.A., Razzak J. (2020). Telemedicine in the United States: An Introduction for Students and Residents. J. Med Internet Res..

[11] Velasquez D., Mehrotra A. Ensuring the Growth of Telehealth during COVID-19 Does Not Exacerbate Disparities in Care. Health Affairs Blog..

[12] Vogels E. Digital Divide Persists Even as Americans with Lower Incomes Make Gains in Tech Adoption. Pew Research Center. Published 22 June 2021. https://www.pewresearch.org/fact-tank/2021/06/22/digital-divide-persists-even-as-americans-with-lower-incomes-make-gains-intech-adoption/.

[13] Eberly L.A., Khatana S.A.M., Nathan A.S., Snider C., Julien H.M., Deleener M.E., Adusumalli S. (2020). Telemedicine outpatient cardiovascular care during the COVID-19 pandemic: Bridging or opening the digital divide?. Circulation.

[14] Scott Kruse C., Karem P., Shifflett K., Vegi L., Ravi K., Brooks M. (2018). Evaluation barriers to adopting telemedicine worldwide: A systematic review. J. Telemed. Telecare.

[15] Addressing Equity in Telemedicine for Chronic Disease Management during the COVID-19 Pandemic. NEJM Catalyst: Innovations in Care Delivery. Published 4 May 2020. https://catalyst.nejm.org/doi/full/10.1056/CAT.20.0123.

[16] Kontos E., Blake K.D., Chou W.-Y.S., Prestin A. (2014). Predictors of eHealth Usage: Insights on The Digital Divide from the Health Information National Trends Survey 2012. J. Med. Internet Res..

[17] Jackson L.A., Zhao Y., Kolenic A., Fitzgerald H.E., Harold R., Von Eye A. (2008). Race, gender, and information technology use: The new digital divide. CyberPsychology Behav..

[18] Telemedicine Use—Household Pulse Survey—COVID-19. Centers for Disease Control and Prevention. Published 19 January 2022. https://www.cdc.gov/nchs/covid19/pulse/telemedicine-use.htm.

[19] U.S Census Bureau Source of the Data and Accuracy of the Estimates for the Household Pulse Survey-Phase 3.2. https://www2.census.gov/programs-surveys/demo/technical-documentation/hhp/Phase3-2_Source_and_Accuracy_Week%2036.pdf.

[20] 5 CFR § 46.117—Documentation of Informed Consent. Cornell Law School. https://www.law.cornell.edu/cfr/text/45/46.117.

[21] Code of Federal Regulations. A Point in Time eCFR System. https://www.ecfr.gov/current/title-45/subtitle-A/subchapter-A/part-46/subpart-A/section-46.117.

[22] United States Census Bureau Phase 3.3 Household Pulse Survey. https://www2.census.gov/programs-surveys/demo/technical-documentation/hhp/Phase3-3_Questionnaire_12_01_21_English.pdf.

[23] Hincapié M.A., Gallego J.C., Gempeler A., Piñeros J.A., Nasner D., Escobar M.F. (2020). Implementation and Usefulness of Telemedicine During the COVID-19 Pandemic: A Scoping Review. J. Prim. Care Community Health.

[24] Truex G. As Telehealth Technology and Methodologies Mature, Consumer Adoption Emerges as Key Challenge for Providers. J.D. Power. Published 2019. https://www.americantelemed.org/resources/telehealth-adoption-and-usage.

[25] Pogorzelska K., Chlabicz S. (2022). Patient Satisfaction with Telemedicine during the COVID-19 Pandemic—A Systematic Review. Int. J. Environ. Res. Public Health.

[26] Monaghesh E., Hajizadeh A. (2020). The role of telehealth during COVID-19 outbreak: A systematic review based on current evidence. BMC Public Health.

[27] Bouabida K., Lebouché B., Pomey M.-P. (2022). Telehealth and COVID-19 Pandemic: An Overview of the Telehealth Use, Advantages, Challenges, and Opportunities during COVID-19 Pandemic. Healthcare.

[28] Fouad A.A., Osman M.A., Abdelmonaem Y.M.M., Karim N.A.H.A. (2023). Awareness, knowledge, attitude, and skills of telemedicine among mental healthcare providers. Middle East Curr. Psychiatry.

[29] Mann D.M., Chen J., Chunara R., Testa P., Nov O. (2020). COVID-19 transforms health care through telemedicine: Evidence from the field. J. Am. Med. Inform. Assoc..

[30] Smrke A., Younger E., Wilson R., Husson O., Farag S., Merry E., Macklin-Doherty A., Cojocaru E., Arthur A., Benson C. (2020). Telemedicine During the COVID-19 Pandemic: Impact on Care for Rare Cancers. JCO Glob. Oncol..

[31] Krysta K., Romanczyk M., Diefenbacher A., Krzstanek M. (2021). Telemedicine treatment and care for patients with intellectual disa-bility. Int. J. Environ. Res. Public Health.

[32] Telehealth and Disability: Challenges and Opportunities for Care. National Health Law Program. Published 6 May 2020. https://healthlaw.org/telehealth-and-disability-challenges-and-opportunities-for-care/.

[33] Stevens J.P., Mechanic O., Markson L., O’Donoghue A., Kimball A.B. (2021). Telehealth Use by Age and Race at a Single Academic Medical Center During the COVID-19 Pandemic: Retrospective Cohort Study. J. Med. Internet Res..

[34] Alsabeeha N.H., Atieh M.A., Balakrishnan M.S. (2022). Older adults’ satisfaction with telemedicine during the COVID-19 pandemic: A systematic review. Telemed. e-Health.

[35] Adams R.B., Nelson V.R., Holtz B.E. (2021). Barriers for telemedicine use among nonusers at the beginning of the pandemic. Telemed. Rep..

[36] Eberly L.A., Kallan M.J., Julien H.M., Haynes N., Khatana S.A.M., Nathan A.S., Snider C., Chokshi N.P., Eneanya N.D., Takvorian S.U. (2020). Patient Characteristics Associated with Telemedicine Access for Primary and Specialty Ambulatory Care During the COVID-19 Pandemic. JAMA Netw. Open.

[37] Kakani P., Sorensen A., Quinton J.K., Han M., Ong M.K., Kamdar N., Sarkisian C.A. (2021). Patient Characteristics Associated with Telemedicine Use at a Large Academic Health System Before and After COVID-19. J. Gen. Intern. Med..

[38] Lam A.C., Berliner B., Barrat V.X. (2021). Students’ Use of School-Based Telemedicine Services and Rates of Returning to Class After These Services in a Small Elementary School District (REL 2021–078).

[39] Campos-Castillo C., Anthony D. (2021). Racial and ethnic differences in self-reported telehealth use during the COVID-19 pandemic: A secondary analysis of a US survey of internet users from late March. J. Am. Med Informatics Assoc..

[40] Jones J., Sullivan P.S., Sanchez T.H., Guest J.L., Hall E.W., Luisi N., Zlotorzynska M., Wilde G., Bradley H., Siegler A.J. (2020). Similarities and Differences in COVID-19 Awareness, Concern, and Symptoms by Race and Ethnicity in the United States: Cross-Sectional Survey. J. Med. Internet Res..

[41] Williams D.R., Wyatt R. (2015). Racial Bias in Health Care and Health: Challenges and Opportunities. JAMA.

[42] Hilbert M. (2011). Digital gender divide or technologically empowered women in developing countries? A typical case of lies, damned lies, and statistics. Women’s Stud. Int. Forum.

[43] Lucas J.W., Villarroel M.A. (2022). Telemedicine Use among Adults: United States, 2021.

[44] Reed M.E., Huang J., Graetz I., Lee C., Muelly E., Kennedy C., Kim E. (2020). Patient characteristics associated with choosing a telemedicine visit vs. office visit with the same primary care clinicians. JAMA Netw. Open.

[45] Frederiksen B., Ranji U., Salganicoff A., Long M. (2021). Women’s Experiences with Health Care during the COVID-19 Pandemic: Findings from the KFF Women’s Health Survey. https://www.kff.org/womens-health-policy/issue-brief/womens-experiences-with-health-care-during-the-covid-19-pandemic-findings-from-the-kff-womens-health-survey/.

[46] USDA ERS—Measurement. Published 2022. https://www.ers.usda.gov/topics/food-nutrition-assistance/food-security-in-the-u-s/measurement/.

[47] Solimini R., Busardò F.P., Gibelli F., Sirignano A., Ricci G. (2021). Ethical and Legal Challenges of Telemedicine in the Era of the COVID-19 Pandemic. Medicina.

[48] U.S. Department of Health and Human Services Telehealth Policy Changes after the COVID-19 Public Health Emergency. https://telehealth.hhs.gov/providers/policy-changes-during-the-covid-19-public-health-emergency/policy-changes-after-the-covid-19-public-health-emergency.

[49] Centers for Medicare and Medicaid Services Medicare Physician Fee Schedule Final Rule Summary: CY 2023. https://www.cms.gov/files/document/mm12982-medicare-physician-fee-schedule-final-rule-summary-cy-2023.pdf.

[50] Kim C. (2020). Telemedicine: Healthcare’s Response to the COVID Crisis is Not without Disadvantages. Biomed. J. Sci. Tech. Res..

